# How the Fewest Become the Greatest. *L. casei’s Impact* on Long Ripened Cheeses

**DOI:** 10.3389/fmicb.2018.02866

**Published:** 2018-11-22

**Authors:** Benedetta Bottari, Alessia Levante, Erasmo Neviani, Monica Gatti

**Affiliations:** Department of Food and Drug, University of Parma, Parma, Italy

**Keywords:** *Lactobacillus casei* group, raw milk, ripened cheese, technological drivers, effect

## Abstract

Members of the *Lactobacillus casei* group, including species classified currently as *L. casei*, *L. paracasei*, and *L. rhamnosus*, are among the most frequently found species in raw milk, hard cooked, long-ripened cheeses. Starting from very low numbers in raw milk, they become dominant in the cheese during ripening, selected by physical and chemical changes produced by cheese making and ripening. Their presence at different stages of cheese making and ripening is crucial in defining product features. For these reasons, the scientific community has been more and more interested in studying these “tiny but mighty microbes” and their implications during cheese making and ripening. The present paper reviews the current literature on the effect of *L. casei* in cheeses, with particular reference to the case of Parmigiano Reggiano and Grana Padano, two of the most famous PDO (Protected Designation of Origin) Italian cheeses. Recent advances regarding the selection of new wild strains able to persist until the end of ripening and carrying out slow but crucial activities resulting in specific aromatic features, are also presented.

## Introduction

*Lactobacillus casei* group species, although initially present in low numbers in raw milk, have been proven to be fundamental for the development of the appreciated aromas of raw cow’s milk long ripened cheeses such as Grana Padano (GP) and Parmigiano Reggiano (PR). Indeed, these “tiny but mighty” microbes have drawn attention of the scientific community to explore the contributions of *L. casei* species to the ripened cheeses features. In this mini-review, this topic is addressed in five sections.

### Who Are They?

**FIGURE 1:**
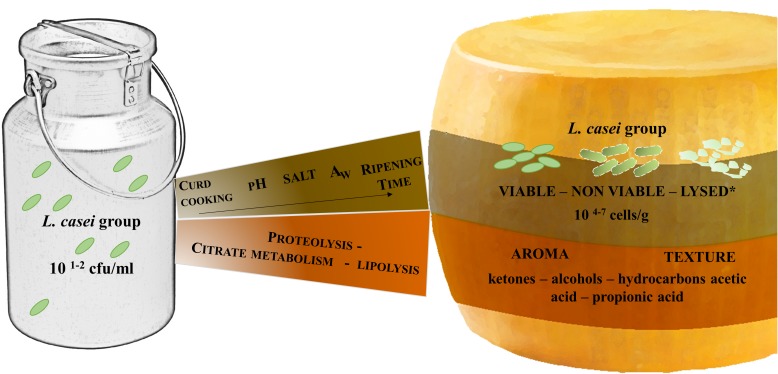
The fate of *L. casei* group from milk to raw cow’s milk, hard cooked, long-ripened cheeses. ^∗^The values reported are retrieved by works of Gatti et al. (2008); D’Incecco et al. (2016); Lazzi et al. (2016).

The *L. casei* group, including the species currently classified as *L. casei*, *L. paracasei*, and *L. rhamnosus*, are among the most frequently found species in hard cooked, long-ripened cheeses (Gala et al., 2008; Gatti et al., 2008, 2014; Monfredini et al., 2012; Solieri et al., 2012; Montel et al., 2014). Thanks to their ability to resist chemical and physical stresses and to use energy sources other than lactose during ripening, strains belonging to these species can tolerate the hostile cheeseenvironment, and represent the dominant microflora of the ripened cheese (Figure 1). Being responsible for the maturation of cheese, rather than taking part in the lactose fermentation, they are usually indicated as non starter lactic acid bacteria (NSLAB) (Settanni and Moschetti, 2010; Gatti et al., 2014).

Several raw milk, hard, cooked, long ripened cheeses are produced in Europe (Gatti et al., 2014), such as Swiss cheeses, Emmental, Gruyere, Sbrinz, Hobelkäse, Comté and West Country Farmhouse Cheddar which are usually ripened for 6 to 48 mo (Depouilly et al., 2004; Harbutt, 2009; Turgay et al., 2011), Scandinavian Västerbottenost, Prästost, Herrgärdsost, Danbo, Jarlsberg cheeses, Low Countries’ Postel, Leisde Kaas, old Edam and old Gouda cheese which are ripened up to 36 mo (Harbutt, 2009). However, probably, the most widely known are the Italian cheeses “Grana Padano” (GP) and “Parmigiano Reggiano” (PR), collectively called grana cheeses. They are Protected Designation of Origin (PDO) cheeses, produced from partially skimmed raw cow’s milk and natural whey starters (Bottari et al., 2010; Santarelli et al., 2013b; Gatti et al., 2014). They share a similar cheese-making protocol, albeit with specific differences, and a very long ripening period of at least 9 and 12 months for GP and PR, respectively, and of up to 20 to 24 mo or longer in the case of gold-label PR cheese (Gatti et al., 2014). The transformation of milk into GP and PR cheese involves a combination of milk, rennet, microorganisms, and salt, which are processed together according to different protocols (Neviani et al., 2013). For both cheeses, the vat milk is heated to 32–34°C and LAB from natural whey starter and calf rennet powder that causes milk coagulation are added. When the curd reaches the proper firmness it is broken down into little granules and heated up to 53–56°C under stirring, whereupon curd granules aggregate at the bottom of the vat (Neviani et al., 2013). The cooking process together with the acidity produced by the starter LAB (SLAB), promotes the formation of the proper texture of curd granules and whey drainage. After curd extraction from the vat, cutting and molding, cheese is immerged in saturated salt brine for about 3 weeks and finally placed in the ripening room (Neviani and Carini, 1994; Mucchetti and Neviani, 2006; Gobbetti et al., 2018b). The described cheese making processes involve dynamic microbial communities of LAB, that have a fundamental role during both cheese-making and ripening, by performing several biochemical reactions, largely based on glycolytic and proteolytic activities (Neviani et al., 2013). During cheese-making, heat load, pH, water activity (a_w_), and redox potential gradually change, inducing a selection of microbial cells. Thus survival, growth, and biochemical activity of microorganisms in cheese are connected to the bacteria stress response to physical and chemical changes in the curd-cheese environment (Neviani et al., 2013; Gobbetti et al., 2018a).

### Where Do They Come From?

NSLAB consist mainly of mesophilic facultative and obligate hetero-fermentative lactobacilli and represent the cheese “*autochthonous*” microbiota, although, in some cheeses, they can also be added as adjunct starters to the milk to supplement the naturally occurring LAB and enhance the development of flavor (Gobbetti et al., 2015). When they are not intentionally added, as for GP and PR, they can come from milk (Addis et al., 2016), dairy environment (Montel et al., 2014), or natural cultures (Gatti et al., 2014). Although raw milk is considered the main source for NSLAB, the link between milk and whey, which is the starting point for natural whey starter preparation, is very close and thus, it is possible to find NSLAB (species not involved in acidification) also in natural whey starter and SLAB (species involved in acidification) also in milk (Neviani et al., 2013). Of course, the specific composition of the milk microbiota directly impacts the subsequent development of dairy products (Quigley et al., 2013), therefore, the use of unpasteurized milk, guarantees the enrichment of microbiota of the cheese making environment (Montel et al., 2014). Raw milk contains a diverse and complex microbial population (Quigley et al., 2011; Vacheyrou et al., 2011; Addis et al., 2016), coming from an endogenous route or colonizing the milk form teat canal, udder skin, milking machines, containers and tanks used for its storage, reflecting the farm and the pasture environment as well (Addis et al., 2016). How many of these biotypes after passing into the milk will be able to multiply and become an important part of milk core microbiota is not easy to understand and not well documented yet. Raw-milk cheeses are considered to taste better than those made from pasteurized milk, indicating that the raw milk microbiota (including NSLAB) and perhaps heat-sensitive enzymes have an effect on flavor (Langford et al., 2012; Neviani et al., 2013). Curd cooking, use of natural whey starters and long ripening time, are the drivers that impact the development of *L. casei* group in cheeses (Gobbetti et al., 2015).

### How Many Are They?

Although it is generally accepted that LAB are one of the dominant group in bovine milk (Quigley et al., 2011, 2013; Montel et al., 2014), *Lactobacillus* genus is only a minority within the complex microbiota (Quigley et al., 2013; Addis et al., 2016; Bancalari et al., 2017). In fact, the total plate count of raw cow’s milk generally ranges from 5 ^∗^ 10^3^ to 10^4^ colony forming units per mL (cfu/mL), with more than 100 genera and 400 microbial species detected, but *Lactobacillus* spp only accounts for about 10^1-2^ cfu/mL (Montel et al., 2014). The isolation frequencies of the different species of lactobacilli in raw milk are quite different, with *L. paracasei* being the most frequently identified species (Vacheyrou et al., 2011). During spontaneous creaming, which occurs both in PR and GP, LAB tend to increase (Carminati et al., 2008) and in the vat milk, the microflora is enriched by SLAB of the whey starter, where NSLAB can be present in low numbers (Coppola et al., 2000; Neviani et al., 2009; Rossi et al., 2012; Pogacic et al., 2013). Within 48 h from when the cheese-making begins, the total LAB count starts to decrease, typically to levels < 10^8^ cfu/g (Santarelli et al., 2013a), with an NSLAB count in acidified curds of about 10^3^ to 10^4^ cfu/g in PR curds (Coppola et al., 2000; De Dea Lindner et al., 2008; Gatti et al., 2008) and GP curds, respectively (Santarelli et al., 2013a). After brining, NSLAB start to grow, reaching cell density of 10^6^–10^8^ cfu/g after several months of aging (Gatti et al., 2014).

Different techniques are available today to study dairy microbial communities by means of culture-dependent and/or culture-independent approaches (Neviani et al., 2013). The culture-dependent approach relies on growing bacteria on suitable media that can easily recover the majority of microorganisms but being too generic and not selective enough to differentiate species which are present in low amounts (Neviani et al., 2013). In particular, Neviani et al. (2009) reported the use of a cheese-based medium (cheese agar medium, CAM). This medium, made up with 24-months old grated cheese, fosters the growth of those microorganisms that better adapt to the changes in nutritional availability and technological parameters occurring during cheese making and ripening (Gatti et al., 2014). CAM has been also reported to improve the recovery of *L. casei* group, particularly *L. rhamnosus* (Neviani et al., 2009). Other authors proposed alternative media for the selective enumeration of *L. casei* in dairy samples (Colombo et al., 2014), however, these methods can still give ambiguous results or present disadvantages such as low reproducibility and time-consuming protocols (Bottari et al., 2017). Culture-independent methods based on bacterial DNA or RNA to identify active members of the microbial population, have been proven to represent a significant tool to follow microbial dynamics in cheeses (Ercolini, 2013; De Filippis et al., 2014). Nevertheless, the discrimination within the *Lb. casei* group by 16S rRNA sequencing, is not possible because of the high similarity in their16S sequence (Felis and Dellaglio, 2007). The monitoring of key genes with significant intra-species heterogeneity has been thus proposed (Felis et al., 2001; Ventura et al., 2003; Watanabe et al., 2008; Huang and Lee, 2011; Huang et al., 2011; Iacumin et al., 2014). Recently, the heterogeneity in the transcribed region of *spxB*, a functional gene conserved in a wide number of *L. casei* group strains, allowed to monitor the active bacterial community involved in different stages of cheese ripening trough RT-qPCR (Savo Sardaro et al., 2016). The importance of identifying wild microorganisms at the species level was mentioned also by (Bottari et al., 2017), who developed a multiplex PCR targeting the gene *mutL*, which could better represent the genome-wide diversity of the three species *L. casei, L. paracasei* and *L. rhamnosus.* However, considerable level of intra-species heterogeneity have been found even between strains isolated from the same niche (Stefanovic and McAuliffe, 2018).

A further step forward is high-throughput sequencing (HTS), a more sensitive method that allows the study of microbial diversity in fermented foods such as cheeses, detecting low abundance species (Ercolini, 2013; De Filippis et al., 2017). The HTS-based monitoring of different strains in the microbiota was proposed by selecting key genes with significant intra-species heterogeneity, and the approach was applied to *Streptococcus thermophilus* (De Filippis et al., 2014; Ricciardi et al., 2016), and was also exploited to monitor the evolution of the metabolically active *L. casei* group during cheese ripening (Levante et al., 2017). More recently, subspecies-specific identification methods based on housekeeping gene sequences and whole-cell matrix-assisted laser desorption/ionization time-of-flight mass spectrometry (MALDI-TOF MS) spectral pattern analysis have been developed to identify members of the *L. casei* group both at the species and subspecies levels (Huang et al., 2018). The possibility to detect different biotypes in specific ripening stages would be extremely interesting, as it could be informative on their potential role and correlate to their peculiar technological properties (Bove et al., 2011).

### What Do They Do?

As already mentioned, *L. casei* group species originates mainly from raw milk and the environment as a result of contamination during the manufacturing procedure, where they’re typically not a dominant species. Differently from SLAB that rapidly decrease after cheese brining, undergoing autolysis and releasing enzymes into the matrix, around 2 month after cheese making, the *L. casei* group species start to slowly grow and increase in number (Gatti et al., 2008). When this occurs, most of the residual lactose in the cheese has already been utilized by the SLAB, suggesting a good adaptation of NSLAB species to unfavorable growth conditions (Succi et al., 2005). The prevalent strains of *Lactobacillus casei* in cheese cores are often different from those found in raw milk, indicating that cheese conditions determine strains selections (Montel et al., 2014).

The ability of *L. casei* species to survive sugar starvation during cheese ripening, has been linked to the use of the nitrogen fraction (amino acids and peptides) and/or products of SLAB lysis as alternative energy sources (Fox et al., 2004; Bove et al., 2011; Sgarbi et al., 2013a).

Thanks to these abilities, *L. casei* group species are among the most frequently found species in ripened PR and GP (Gala et al., 2008; Gatti et al., 2008; Monfredini et al., 2012; Solieri et al., 2012). The adaptation of NSLAB to the cheese environment is a complex process. Bove et al. (2012) demonstrated that, compared with cultivation on a synthetic rich medium, *L. rhamnosus* strains grown under cheese-like conditions, increased the amount of proteins responsible for citrate catabolism, acetate production, proteolytic activity and amino acid catabolism, while decreasing the amount of proteins responsible for sugar transport, glycogen biosynthesis, pentose phosphate pathway, EPS biosynthesis, and cell wall biosynthesis. Of course, NSLAB in cheese represent a dynamically evolving microorganisms’ population, where different cells are growing, being dormant or even dying. Interestingly, a recent study has shown that cells of *L. rhamnosus* cultivated under cheese-like conditions expresses a type I toxin-antitoxin system that might led the bacteria in a dormant state; furthermore, expression of the genes encoding for the novel system, named *Lpt*, was detected in cheeses, at 6 and 12 months of ripening (Folli et al., 2017). Also, NSLAB lysis has been proven after 2 months of ripening in GP (Santarelli et al., 2013a) and 6 months of ripening in PR (Gatti et al., 2008), thus their contribute to cheese flavor and aroma formation depends also on enzymes released after cells lysis (Smid and Kleerebezem, 2014).

### What’s Their Effect?

*Lactobacillus casei* group species play a key role in cheese ripening by multiplying after cheese brining and by releasing enzymes into the cheese later in ripening (Gatti et al., 2008, 2014). The metabolism of NSLAB growing under hostile conditions leads to products involved in cheese flavor characterization (Gobbetti et al., 2015; Lazzi et al., 2016). Viable cells and enzymes released by lysed cells work together during cheese ripening resulting in biochemical reactions that generate compounds responsible for cheese flavor (Smid and Kleerebezem, 2014). In particular, three major LAB metabolic pathways are involved in flavor formation: (i) metabolism of lactate and citrate, (ii) proteolysis and subsequent amino acid catabolism, and (iii) release of free fatty acids and their subsequent metabolism (McSweeney and Sousa, 2000; Yvon and Rejien, 2001). Peptides and aminoacids, increasing along with the ripening progress, constitute the main nutritional compounds for NSLAB (Fox et al., 2004; Settanni and Moschetti, 2010; Settanni and Moschetti, 2010). *L. rhamnosus* strains, as an example, have been linked to arginine catabolism in ripened cheeses (D’Incecco et al., 2016), and the metabolism of the sulfur-containing amino acids cysteine and methionine has been described for one *Lactobacillus paracasei* strain (Wüthrich et al., 2018) However, NSLAB can also utilize carbohydrates deriving from glycomacropeptides of caseins, glycoproteins from fat globule membranes, pentoses and other products from SLAB lysis and residual citrate (Fox et al., 2004; Sgarbi et al., 2013a). Through citrate metabolism, acetoin is produced, while by degradation of amino acids produces other ketones that are considered common constituents of cheese aroma (McSweeney and Sousa, 2000; Curioni and Bosset, 2002; Marilley and Casey, 2004). The metabolic potential of the *L. casei* group can vary according to the species and has been found to be differently expressed at strain level (Stefanovic et al., 2017b). Indeed, the metabolic activity of *L. casei* and *L. rhamnosus* has been linked to the presence of higher amounts of ketones and acetic acid in ripened cheeses such as PR and GP (Bove et al., 2012; Sgarbi et al., 2013b; Lazzi et al., 2014). One *L. paracasei* strain producing high amount of 3-methyl-butanoic acid, 2-methyl-propanoic acid, diacetyl, acetoin, 2,3-butanediol and ethanol, related to a pleasant buttery/creamy or strong cheese aromatic notes, was isolated from raw milk used for the production of PR cheese (Bancalari et al., 2017). In the same study, from the same milk, one (putative) *L. casei* strain producing 3-methylbutanal and 3-methyl-1-butanol, and two *L. paracasei* strains producing 1-hexanol, were isolated. These compounds are related, respectively, to fruity/green/nutty notes and fruity/flower aromatic notes, which positively affect the overall flavor perception of ripened cheeses. A GP cheese characterized by a complex microbial composition, where *L. rhamnosus/L. casei* grew during ripening, had a high complexity of volatile compounds. In particular, greater amounts of ketones, alcohols, hydrocarbons, acetic acid and propionic acid were revealed and correlated mainly with the presence of the *L. casei* group species (Lazzi et al., 2016).

## Concluding Remarks and Future Perspectives

One of the reason for the delightfulness of two of the most famous PDO Italian cheeses can be closely related to the effect of the presence, development and lysis of the *Lactobacillus casei* group strains naturally present in raw milk (Gobbetti et al., 2018a). Considering this, it is clear that the current knowledge brought us to the starting point for research on new wild strains with specific aromatic features. Recently, an original approach has been proposed to isolate, directly from milk, *L. casei* group strains to be potentially used as adjunct starter to improve the taste of new cheeses (Bancalari et al., 2017), and a screening approach for the knowledge-based selection of strains potentially enabling flavor diversification in cheeses has been provided (Stefanovic et al., 2017a). Also, the identification of mechanism for bacterial adaptation and persistence in the *L. casei* group, such as toxin-antitoxin modules, opens a new path to investigate the molecular basis of bacterial adaptation to cheese (Folli et al., 2017). The still largely unexplored field of non growing but metabolically active bacterial cells in food environments potentially offer a new perspective to understand how and why certain strains belonging to the species of *L. casei* group are capable, once they have entered the cheese manufacturing process, to persist to “technological” stresses in viable form until very long ripening times, possibly carrying out a slow but crucial activity on cheese ripening.

## Author Contributions

BB and MG conceived the presented idea. MG designed the paper structure. BB wrote the manuscript with support from MG and AL. AL designed the figure. EN supervised and critically revised the project. All authors discussed the literature and contributed to the final manuscript.

## Conflict of Interest Statement

The authors declare that the research was conducted in the absence of any commercial or financial relationships that could be construed as a potential conflict of interest.
